# Eight ways to stay healthy after cancer: an evidence-based message

**DOI:** 10.1007/s10552-013-0179-z

**Published:** 2013-03-12

**Authors:** Kathleen Y. Wolin, Hank Dart, Graham A. Colditz

**Affiliations:** Division of Public Health Sciences, Washington University School of Medicine and Alvin J. Siteman Cancer Center, 660 S Euclid Ave, Box 8100, St Louis, MO 63110 USA

**Keywords:** Lifestyle, Smoking, Obesity, Exercise, Diet, Survivorship

## Abstract

**Purpose:**

Since 1999, in conjunction with the internationally known and award-winning Your Disease Risk (yourdiseaserisk.org) risk assessment tool, the “Eight Ways to Stay Healthy and Prevent Cancer” message campaign has provided an evidence-based, but user-friendly, approach to cancer prevention. The scientific evidence behind the campaign is robust and while not a complete list, provides a great deal of benefit in the reduction of cancer risk. With 12 million cancer survivors in the United States, there is a need for a parallel set of recommendations that oncologists and primary care providers may routinely use for individuals following a cancer diagnosis focused on improving the quantity and quality of life after diagnosis. With increasing survival rates and many cancer survivors dying from noncancer causes, survivorship care necessarily focuses on more than just risk of cancer recurrence and cancer-related mortality.

**Methods:**

To provide a foundation for living a healthy life after a cancer diagnosis, we developed a set of evidence-based health messages for cancer survivors. “Cancer Survivors’ Eight Ways to Stay Healthy After Cancer,” published by the Siteman Cancer Center at Washington University School of Medicine and Barnes Jewish Hospital, documents both the evidence supporting the recommendations as well as tips for implementing them.

**Results:**

The one-line summary messages are: (1) don’t smoke, (2) avoid secondhand smoke, (3) exercise regularly, (4) avoid weight gain, (5) eat a healthy diet, (6) drink alcohol in moderation, if at all, (7) stay connected with friends, family, and other survivors, (8) get screening tests and go to your regular checkups.

**Conclusions:**

The cancer survivors’ eight ways are the foundation for an evidence-based health promotion program for survivors.

Since 1999, in conjunction with the internationally known and award-winning Your Disease Risk (yourdiseaserisk.org) risk assessment tool, the “Eight Ways to Stay Healthy and Prevent Cancer” message campaign has provided an evidence-based, but user-friendly approach to cancer prevention. The scientific evidence behind the campaign is robust and while not a complete list, provides a great deal of benefit in the reduction of cancer risk [1, 2].

With nearly 12 million cancer survivors in the United States [3], there is a need for a parallel set of recommendations that oncologists and primary care providers may routinely use for individuals following a cancer diagnosis, focused on improving the quantity and quality of life. Nearly, 65 %of cancer survivors live 5 or more years after diagnosis and 5-year survival rates exceed 95 %for two of the most common cancers, breast and prostate [3]. Survivors are at increased risk for second malignancies, cardiovascular disease, diabetes, and osteoporosis [4–7]. Furthermore, cancer survivors die from noncancer causes [1, 4, 5, 8]. Thus, survivorship care necessarily focuses on more than just risk of cancer recurrence and cancer-related mortality and functional limitations [1, 5]. Data suggest that the majority of cancer survivors have at least one comorbid condition [1, 3, 9–12]. Despite the evidence for the benefits of maintaining a healthy lifestyle after a cancer diagnosis, only 20 % of oncologists provide guidelines on lifestyle issues [3–7, 9, 13]. Cancer survivors are also subject to long-term and late effects of treatment and diminished quality of life. The cancer survivors’ eight ways provides a foundation for living a healthy life after a cancer diagnosis.

“Cancer Survivors’ Eight Ways to Stay Healthy After Cancer” (Fig. 1), published by the Siteman Cancer Center at Washington University School of Medicine and Barnes Jewish Hospital, documents both the evidence supporting the recommendations as well as tips for implementing them. Successful implementation of survivorship programming is necessary to improve patient outcomes [3–7, 13, 14]. The one-line summary messages are as follows:

**Fig. 1:**
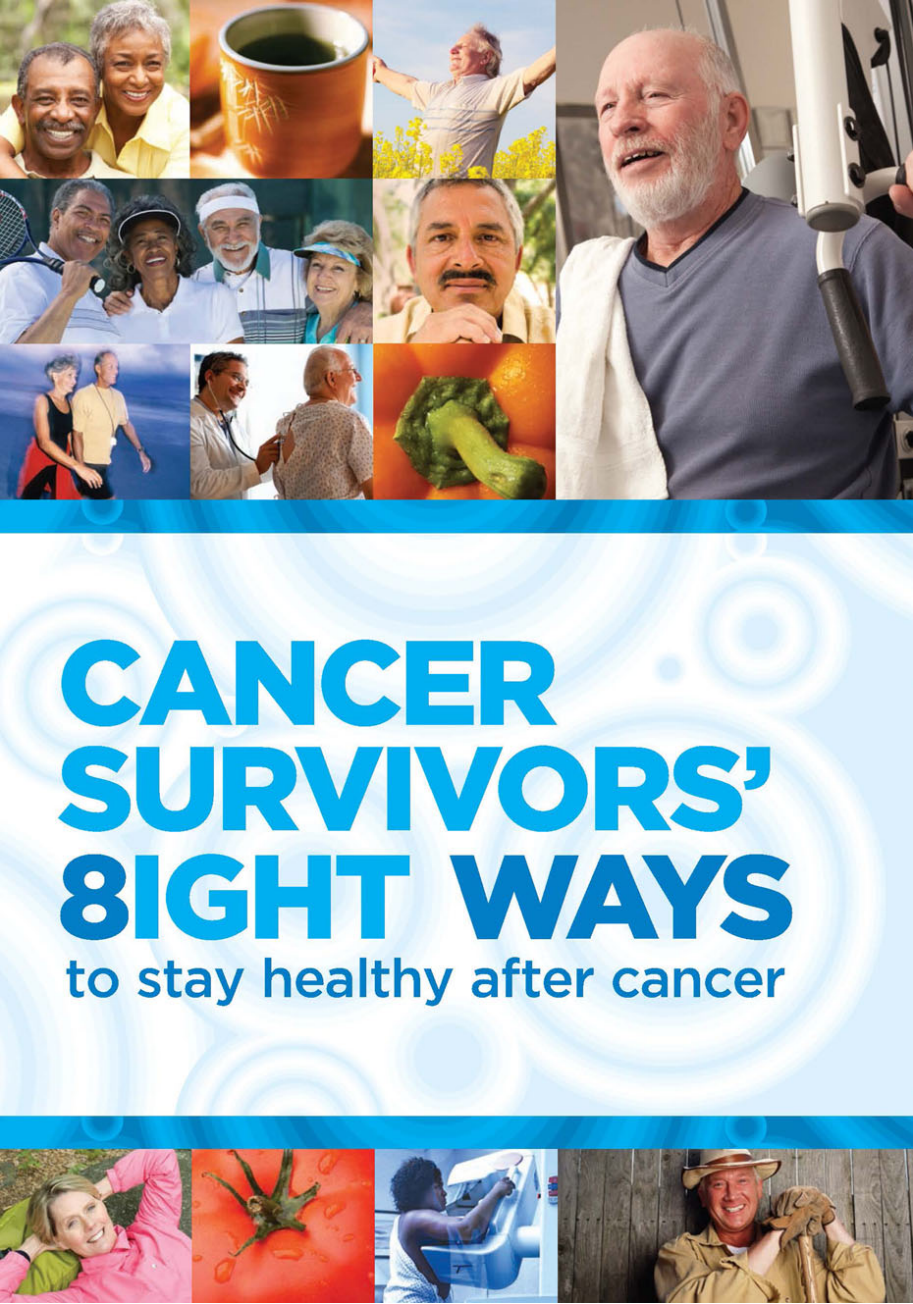

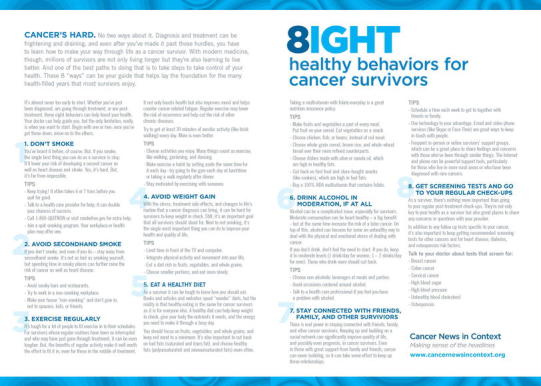

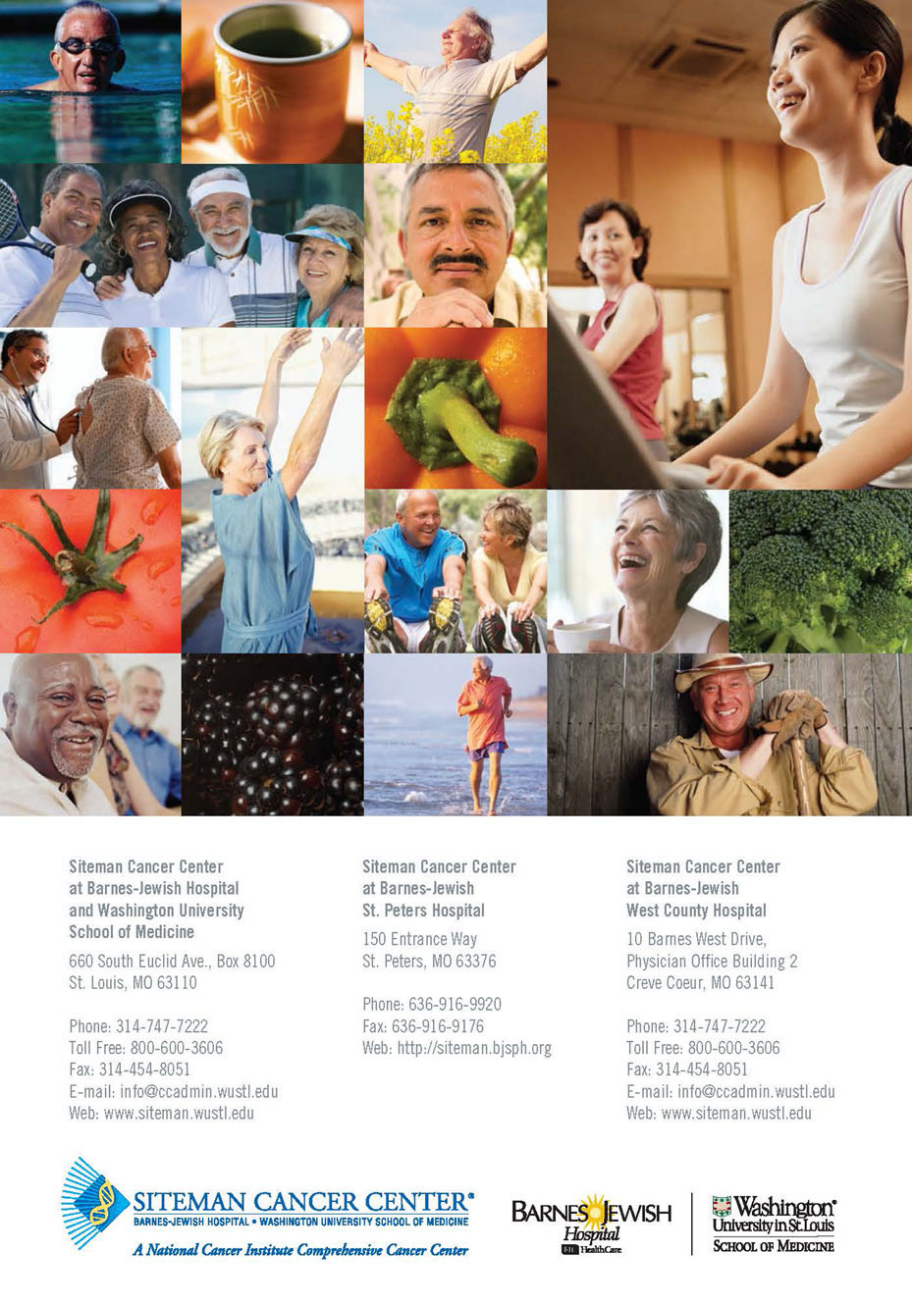
Cancer Survivors’ Eight Ways to Stay Healthy After Cancer. Educational materials from the Siteman Cancer Center at Barnes Jewish Hospital and Washington University School of Medicine. Available at www.cancernewsincontext.org

1. Don’t smoke
2. Avoid secondhand smoke
3. Exercise regularly
4. Avoid weight gain
5. Eat a healthy diet
6. Drink alcohol in moderation, if at all
7. Stay connected with friends, family, and other survivors
8. Get screening tests and go to your regular check-ups.

The messages contained in the review were developed by the authors, building on our epidemiologic, behavioral medicine and health communication expertise and our previous work in cancer prevention and based on our review of the epidemiologic and behavioral medicine literature in cancer survivorship. As this was not a scientific document, a formal systematic literature review was not done. During the course of developing the guidelines and brochure, two authors (GAC, KYW) presented the guidelines at several community-based events, and this feedback was used to make minor modifications to the language used. The published brochures are available in Siteman Cancer Center clinic and patient education areas across institution locations, and the content is also available on the institution website. As such, patients can access it at numerous points in their care, including at diagnosis, during treatment or during posttreatment follow-up and surveillance visits. A formal evaluation of patient or provider perceptions has yet to be conducted.

Here, we review the evidence base behind the cancer survivor’s eight ways. (For the purposes of our report, we employ the National Coalition for Cancer Survivorship definition of survivor, from the point of diagnosis onward [4–8, 14, 15].) Figure 1 provides an action plan for implementing counseling recommendations. We also provide references to evidence-based interventions in survivor populations for the recommendations when available. The body of evidence specifically evaluating interventions in this population is rapidly expanding.

## Don’t smoke

While the original eight ways for cancer prevention focused on the risks of developing cancer and mortality associated with smoking, cancer survivors are subject to their own set of smoking-related risks [4, 5, 8, 15–18]. Survivors who smoke have increased risk of mortality, recurrence, and second primary tumors [5, 16–22]. Smoking also increases treatment-related complication rates [19–24] and reduces quality of life among survivors [23–26]. Finally, smoking is associated with increased risk of cardiovascular disease, stroke, and osteoporosis [15, 25, 26], important comorbidities among cancer survivors.

Smoking cessation increases survival, lowers risk of new primary tumors and metastases, as well as tumor recurrence [15]. Cessation also improves treatment response and reduces treatment toxicities [15, 27].

Despite the harms associated with smoking among cancer survivors, the prevalence of smoking among survivors remains similar to that of the US general population and is higher among young adult survivors than older survivors [27–29] and may be higher than among their nonsurvivor peers [28–30]. Smoking rates among survivors vary by diagnosis [30–32].

Evidence does indicate that smoking cessation interventions in cancer survivors are successful [31–33]. Yet, the evidence of the harms, provision of clinical practice guidelines, and an ability to intervene have not led to regular provision of cessation counseling by providers [13, 33]. Interventions are not well disseminated or integrated into clinical care [13, 34], though some dissemination studies are underway [34, 35]. It is estimated that less than half of cancer centers have a designated tobacco treatment provider [35, 36].

## Avoid secondhand smoke

Given the particular importance for survivors of remaining smoke-free, and the known associations of smoke-free homes with successful quitting, living (and working) in a smoke-free environment is key for survivor health. Data suggest that exposure to secondhand smoke significantly increases the odds of survivors of continuing to smoke and of reporting poor health regardless of their own smoking status [36, 37]. Secondhand smoke is also associated with increased risk of many chronic diseases, including heart disease and stroke [37, 38].

## Exercise regularly

The 2010 American College of Sports Medicine (ACSM) Exercise Guidelines for Cancer Survivors concluded that exercise is safe for cancer survivors during and after treatment [38, 39]. Exercise is associated with myriad benefits among survivors [39–42]. While high-quality observational data have shown exercise reduces risk of recurrence and mortality [38–43], a wealth of intervention trials have shown exercise has more proximal benefits, including improving quality of life, reducing cancer-related fatigue, reducing depressive symptoms, and preventing or minimizing treatment-related side effects [38, 39, 43, 44]. Exercise also reduces risk of diabetes and heart disease [44]. For most cancer survivors, the ACSM guidelines parallel the US Physical Activity Guidelines for Americans [44, 45], advocating that, first and foremost, survivors avoid inactivity, progressing as tolerated to 150 min a week of aerobic exercise with complementary flexibility and resistance training. While it can initially seem counterintuitive, exercise is also a recommended treatment for cancer-related fatigue [45]. Despite the wealth of data supporting the benefits of exercise, several challenges to widespread program implementation have been identified [1, 11, 13, 39]. Community-based programs targeted to cancer survivors are growing, such as the programs provided at YMCA’s across the country in partnership with LIVESTRONG (http://www.livestrong.org/What-We-Do/Our-Actions/Programs-Partnerships/LIVESTRONG-at-the-YMCA/LIVESTRONG-at-the-YMCA-Locations).

## Avoid weight gain

As with exercise, excess weight is a known cancer risk factor but has also been shown to drive outcomes after diagnosis, including recurrence, survival, quality of life, and side effects treatment, such as for lymphedema [13, 46, 47, 54, 55, 56]. Weight at diagnosis is the strongest lifestyle predictor of disease and quality of life outcomes in women with breast cancer [48]. Regardless of baseline weight, weight gain after diagnosis also significantly increased the risk of mortality among breast cancer survivors [49]. In prostate cancer, obesity at the time of diagnosis is associated with an elevated risk of prostate specific antigen (PSA) failure [50]. In colon cancer, limited data suggest that class II and III obesity may increase the risk of mortality and recurrence in survivors [50–52, 53]. Risk of both cardiovascular disease and diabetes increases with weight and weight gain as well.

Balanced against the risk associated with excess weight is the knowledge that cancer treatment can result in unintended weight loss and malnourishment. In these individuals, additional weight loss poses problems, decreasing quality of life, interfering with treatment adherence, and increasing complication risk.

Despite the difficulty of losing weight, several interventions have shown promise and delivered significant changes in weight among cancer survivors, including both face-to-face [57, 58] and distance-based interventions employing print and phone modalities[59, 60]. However, maintaining the weight loss remains a challenge in cancer survivors [61].

## Eat a healthy diet

Many individuals come to a cancer diagnosis with a history of unhealthy eating habits, but the point of diagnosis provides an opportunity to make dietary choices that improve health. As with physical activity and obesity, some of the benefits to a healthy diet in cancer survivors come from the reduction in risk of other chronic diseases, including diabetes and heart disease [62]. However, evidence also points to direct cancer-related benefits to a healthy diet.

The Women’s Intervention Nutrition Study (WINS) randomized women with early-stage breast cancer to a low-fat diet and found a significant reduction in risk of recurrence [63]. However, the women on the low-fat diet also lost weight, which may confound the dietary findings. In contrast, the Women’s Healthy Eating and Living (WHEL) study found no effect of a high fiber, fruit, and vegetable and low-fat diet on recurrence among breast cancer survivors [64]. Despite the known benefits of a healthy diet in preventing cancer [65, 66], few specific diet components have been evaluated in cancer survivors. Data from the Nurses’ Health Study showed a significant reduction in mortality among breast cancer survivors adhering to a diet high in fruits, vegetables, legumes, whole grains, poultry, and fish [67]. Earlier analyses in this population suggested no effect of a low-fat diet [68], but a more recent report indicated dietary fat intake after diagnosis increases mortality risk in women with breast cancer [69]. Similar results have been seen in other observational studies that evaluated dietary patterns [70–72]. A high-fiber diet was nonsignificantly associated with reduced risk of mortality in a cohort of breast cancer survivors [73]. Observational data have also linked dietary components to reduced risk of recurrence for men with prostate cancer, specifically intake of fish, tomato sauce, and monounsaturated fats [72, 74]. Fruit and vegetable consumption is also associated with improved quality of life, physical, and cognitive function in survivors [54, 75].

Eating a healthy diet should not be confused with the intake of dietary supplements, which have shown little or no benefit for prognosis after cancer and may in fact result in worse outcomes [76]. The exception to this is intake of vitamin D and calcium, which are recommended to preserve bone health in those who have undergone hormone therapies [77]. As a result, both the American Cancer Society (ACS) [78] and the World Cancer Research Fund/American Institute for Cancer Research have advocated meeting dietary needs through food [65].

WHEL and WINS both demonstrated that interventions in cancer survivors can demonstrate significant dietary changes. Other studies have also had success in improving the diets of survivors. For example, in a 10-month tailored print material intervention, FRESH START significantly decreased saturated fat and improved fruit and vegetable consumption as well as overall diet quality and maintained those changes 2 years after the study started [79, 80].

## Drink alcohol in moderation, if at all

The dual health effects of alcohol make concise pubic health recommendations a challenge, even more so for cancer survivors. There is good evidence that moderate alcohol consumption lowers the risk of cardiovascular disease [81, 82], but alcohol also increases cancer risk. In cancer survivors, alcohol consumption has been linked to worse prognosis [83]. However, with half of deaths in cancer survivors attributable to cardiovascular disease [76], there is a need to balance risks and benefits. Because of the potential harms, nondrinkers should not start drinking to reduce cardiovascular disease risk and should turn to other lifestyle strategies. Those who drink moderately need not stop. Heavy drinkers should cut back to moderate levels or stop drinking altogether. Alcohol consumption may vary widely across diagnoses. One study of testicular cancer survivors [84] noted that over 75 %of those surveyed reported regular alcohol consumption, while in a study of colorectal cancer survivors reported that only 8 %were heavy drinkers [75].

## Stay connected with friends, family, and other survivors

Maintaining social support from friends and family is an evidence-based approach to improving quality of life after a cancer diagnosis. Social support, including that obtained through formal support groups, has been shown to reduce stress, depression, and fatigue in cancer survivors and lead to improvements in mood, self-image, and global quality of life [85–89]. While social support has not been shown to reduce risk of recurrence or mortality [85, 90–92], the quality of life benefits are real and meaningful. Social support also facilitates other positive behavioral changes, such as increasing physical activity [93].

## Get screening tests and go to your regular check-ups

While many cancer survivors receive recommendations for ongoing surveillance of their cancer recurrence risk, preventive testing related to other causes of disease and death can lag. A recent report indicated that prostate cancer survivors were less likely than noncancer controls to be compliant with colorectal cancer screening [94]. Among colon cancer survivors, nearly all (97 %) were compliant with colonoscopy, but 20 % were not compliant with mammography and 37 % were not compliant with Pap smear [95]. Similarly, a national study found the majority of cancer survivors had multiple cardiovascular disease risk factors and more than a third were at high cardiac risk [8]. Such findings highlight the need not just for cancer screening but also for cardiovascular risk factor screening, diabetes screening, and even osteoporosis screening. Together, this indicates a need for regular noncancer care to ensure cancer survivors lead healthy lives engaging in behaviors that reduce their risk of disability and death.

## The need for implementation

Unfortunately, the gaps in survivorship care are well-established [13, 96]. The result of the gaps in care is that lifestyle management of cancer-related endpoints is also not adequately addressed. Most cancer survivors do not follow the cancer prevention guidelines [11, 65, 66] that have existed for some time [3, 97]. Yet, survivors do make behavior changes and are more likely to make positive than negative changes [3, 98]. Thus, the challenge is implementing survivorship programming that directs survivors to the lifestyle behavior choices most likely to benefit them [4–7, 13, 39].

Implementation of lifestyle interventions is subject to the same challenges that cancer care faces as survivors transition from active treatment to ongoing disease monitoring and management. These were detailed extensively in the Institute of Medicine report on the challenges in cancer survivor care [99] and include not just patient challenges, but those faced by providers and institutions. Similarly, Stanton details some of the key challenges to implementing psychosocial care for quality of life after treatment, including the heterogeneity of patient trajectories, the transition from the treatment to posttreatment phase, long-term effects that differ from those in the period more proximal to treatment, and demographic differences in physical and psychosocial outcomes [100].

Implementation of lifestyle interventions may be subject to additional burdens as they occur outside of treatment facilities, are often not covered by insurance for those who have it, and often lack support outside of urban areas and major medical centers. Fortunately, many aspects of the eight ways for cancer survivors can be implemented without expensive facilities or medical support. The brochure in Fig. 1 provides a starting point for initiating this counseling. For example, choosing a whole food diet based on fruits, vegetables, whole grains, and lean proteins can be done anywhere. Most cancer survivors can engage in walking, starting with a low dose and low intensity and progressing as tolerated to meet recommendations. Phone and Internet-based supports exist for smoking cessation and weight management through websites like www.becomeanex.org. Evidence suggests that a physician recommendation for change is associated with an increased likelihood of behavior change adoption among cancer survivors [4, 8, 101]. The implementation of these guidelines, like those for prevention, can also be considered in the broader context of shared decision-making [102].

## Conclusion

The cancer diagnosis often provides a moment of opportunity to consider lifestyle choices as survivors seek guidance from clinicians on what lifestyle choices are most beneficial. In a time where both high- and low-quality health information are readily available through the Internet- and nonevidence-based sources, patients benefit from simple messages grounded in a strong scientific evidence base. It was this premise that led to the expansion of the “Eight Ways to Stay Healthy and Prevent Cancer” to the new “Cancer Survivors’ Eight Ways to Stay Healthy After Cancer.” Reflecting the secular trend toward growing interest in survivorship health, several similar guidelines were developed and released at this time, including the American Cancer Society’s (ACS) Nutrition and Physical Activity Guidelines for Cancer Survivors, a report intended to provide health care providers with information to support informed choices among survivors and their families [103]. Both the ACS guidelines and ours emphasize the importance of exercise and weight management, but differ in the level of attention given to diet, tobacco, and social support. These recommendations provide a starting point for engaging in lifestyle counseling and can be used by patients, providers, cancer centers, and community support groups to focus on strategies most likely to yield benefit with minimal risks and side effects.
